# Epilepsy in patients undergoing cardiac surgery with ExtraCorporeal Circulation: case series and description of a peculiar clinical phenotype

**DOI:** 10.1186/s12883-022-02665-7

**Published:** 2022-04-11

**Authors:** Matteo Pugnaghi, Francesco Cavallieri, Mauro Zennaro, Marialuisa Zedde, Romana Rizzi, Davide Gabbieri, Franco Valzania

**Affiliations:** 1 Neuromotor & Rehabilitation Department, Neurology Unit, Azienda USL-IRCCS Di Reggio Emilia, Viale Risorgimento 80, 42123 Reggio Emilia, Italy; 2 Clinical and Experimental Medicine PhD Program, University of Modena and Reggio Emilia, Modena, Italy; 3 Cardiology Unit, Azienda Ospedaliero-Universitaria Di Modena, Modena, Italy; 4 Cardiac Surgery Division, Hesperia Hospital, Modena, Italy

**Keywords:** Cardiac surgery, Epilepsy, ExtraCorporeal Circulation, Microbleeds, Seizures

## Abstract

**Background:**

Extracorporeal circulation (ECC) is now being increasingly used in critical care settings. Epileptic seizures are a recognized but under reported complication in patients receiving this care. Acute symptomatic post-operative seizures have been described, as well as remote seizure, mostly in the form of convulsive seizures. Epilepsy has also been reported, although with lower frequency and mainly with convulsive seizures, while different seizure semiology is rarely described.

**Case presentation:**

We report a case series of four patients developing epilepsy with homogeneous features following heart surgery with ECC. We present neurophysiological and neuroradiological data and we describe the peculiar characteristics of epilepsies in terms of seizure semiology, frequency, and drug response. The main features are: an insulo-temporal or parieto-occipital semiology, often multifocal and without loss of consciousness or motor manifestations, a high frequency of seizures but with low impact on daily life, and a good response to anti-epileptic therapy.

**Conclusions:**

We hypothesize a pathogenetic mechanism and we discuss the clinical implications of identifying these forms of epilepsy which tend to be often under-recognized.

## Background

Interventional cardiac procedures and cardiac surgery may lead to neurologic complications including seizures, encephalopathy, delirium, stroke and cognitive impairment, that account for significant morbidity and mortality [1]. Even if clinically manifest strokes are rare, studies that have employed postoperative systematic neurological assessments have found a much higher rate of them (17%) [2]. In addition, studies based on perioperative magnetic resonance (MRI) with diffusion-weighted imaging (DWI) sequences have shown the possible presence of cerebral infarction after endovascular procedures even in the absence of a clinically manifest stroke [3]. This is particularly relevant in the setting of cardiac surgery with ExtraCorporeal Circulation (ECC) in which, despite the possibility of controlling the mean arterial pressure, a high incidence of new ischemic lesions has been reported [4]. Clinically evident seizures occur rarely (1–4% of patients) after cardiac surgery, mostly in the form of peri-operative generalized tonic–clonic seizures [5]. However, it should be kept in mind that other types of seizures have been observed after cardiac surgery and that a great proportion of seizures in critically ill patients is nonconvulsive [6]. These seizures are frequently missed or mistaken for different clinical events, leading to the suspicion that their true incidence is probably under-reported [6]. Depending on the type and duration of seizures, they can have long-term consequences, including alteration of neuronal networks, neuronal injury up to neuronal death [7]. Compared to these observations, little data is available for the occurrence of seizures or epilepsy during the long-term follow-up. Here, we report a case series of four patients developing epilepsy with homogeneous features following a heart surgery with ECC.

## Cases presentation

### Case 1

**Fig. 1:**
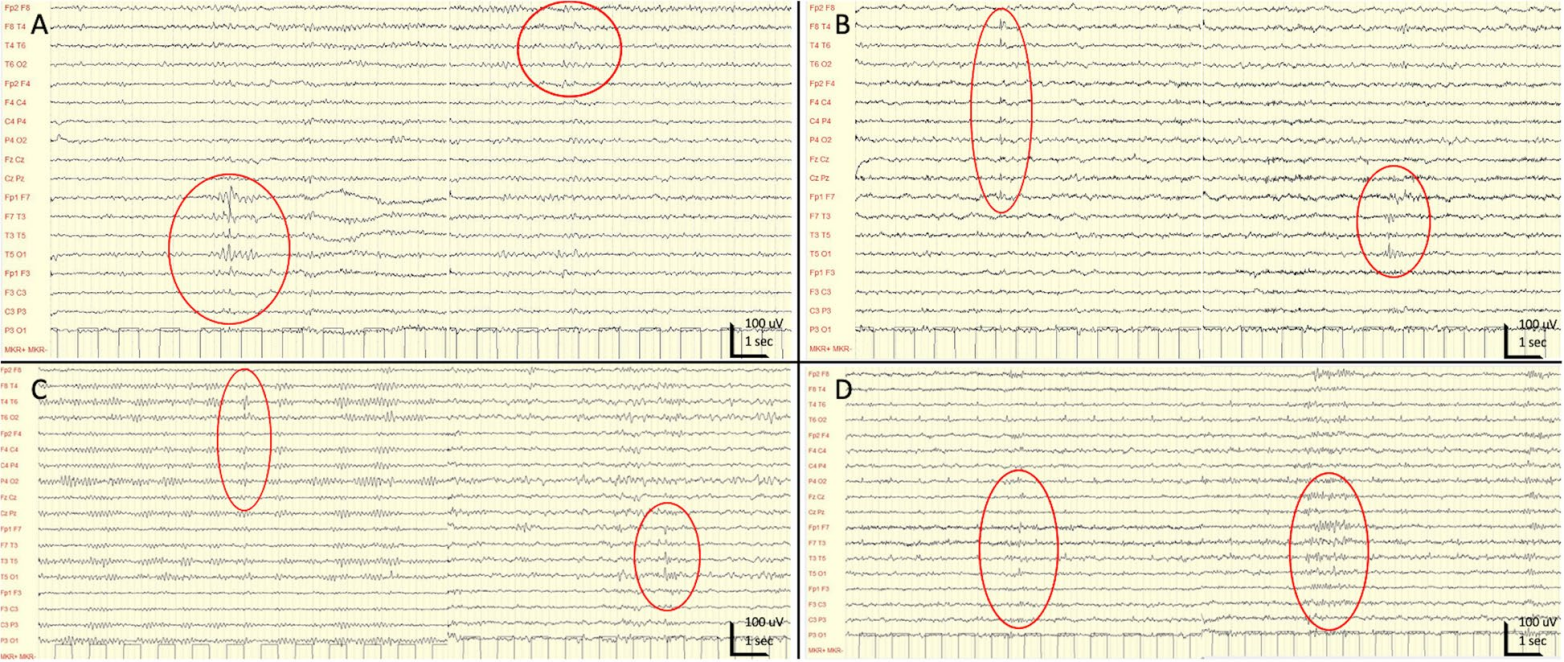
Patients’ EEG. Figure 1 shows the EEGs of the four patients included in this case series. Patient 1 (**A**): the EEG during sleep (NAP) showed asynchronous spikes and slow waves both in right parieto-occipital derivations and in left fronto-temporal region (red circles). Patient 2 (**B**): a NAP EEG showed spikes during sleep in right fronto-central-temporal derivation, and independent slow and sharp waves in left fronto-temporal derivations (red circles). Patient 3 (**C**): NAP EEG revealed sharp waves in right parieto-occipital region and independent spikes localized in left fronto-temporal derivations (red circles). Patient 4 (**D**): the NAP EEG showed sharp waves and spikes in the left fronto-parietal derivations during drowsiness and sleep (red circles)

**Fig. 2:**
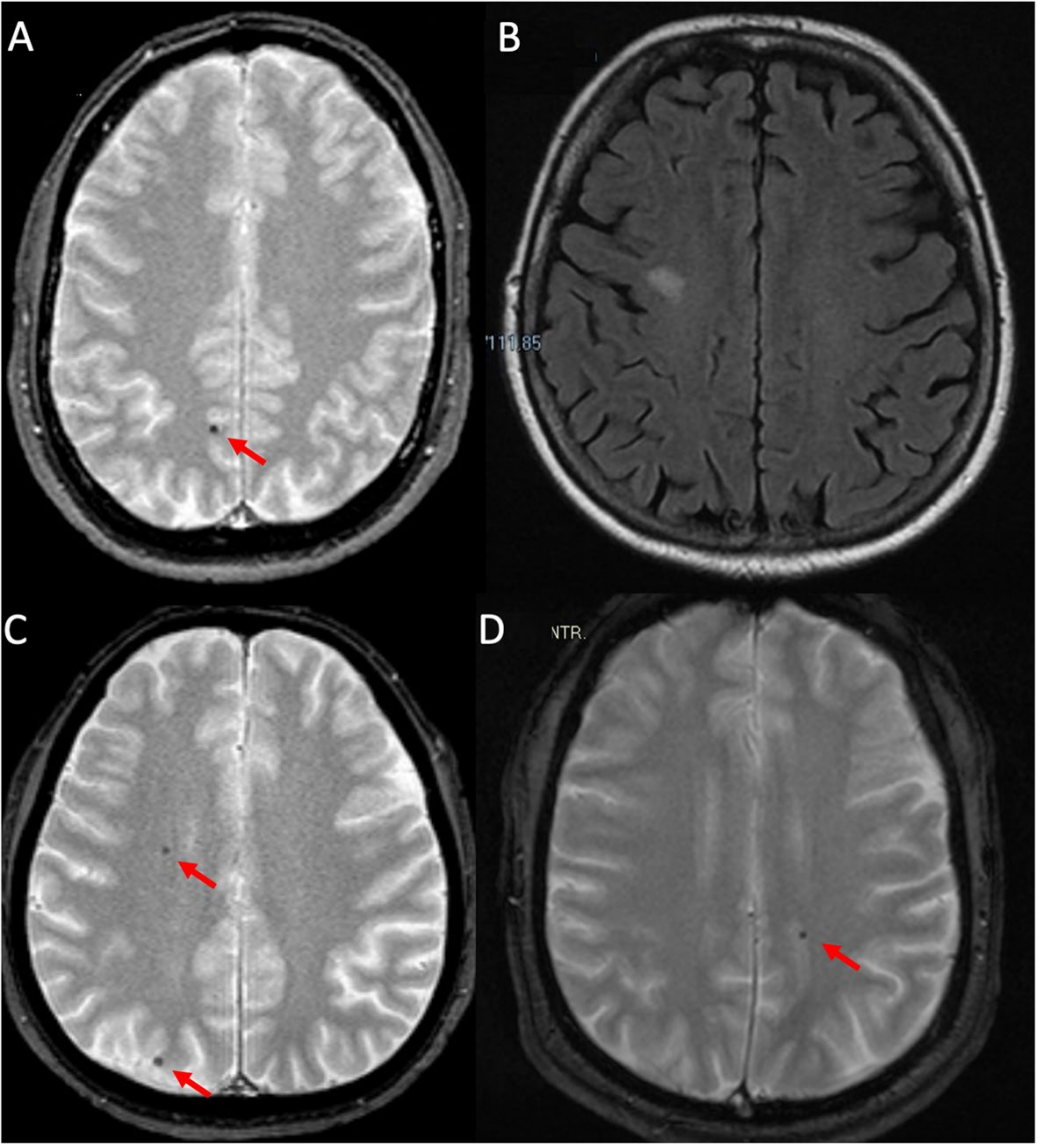
Brain-MRIs. Figure 2 shows brain MRI of the four patients included in this case series. Patient 1 (**A**): brain MRI revealed small microbleeds (MBs), the greater of which was localized in right precuneal region (red arrow). Patient 2 (**B**): brain MRI revealed slight signs of chronic ischemic vascular suffering. Patient 3 (**C**): brain MRI showed small MBs in the right frontal region and right occipital pole (red arrows). Patient 4 (**D**): brain MRI revealed small MB in the left post-rolandic parietal region (red arrow). For other MRI findings see Tab 1

A 57-year-old man underwent, in 2015, a heart surgical intervention of mitral valve reparation and ring annuloplasty with ECC. A week after surgery he started exhibiting episodes characterized by: bitter taste and perioral plus distal left hand fingers paresthesias, and different episodes with visual blackening in the left upper visual field quadrant, without interruption of ongoing activities. The duration was generally a few seconds, with a maximum of 1–2 min, the frequency was initially weekly, and progressively became monthly. After six months following cardiac surgery with ECC, the patient underwent an MRI of the brain which demonstrated few microbleeds (MBs), the greatest of which was localized in the right precuneal region (Fig. 2, A). The electroencephalogram (EEG) during sleep (NAP) showed asynchronous spikes and slow waves both in the right parieto-occipital derivations and in the left fronto-temporal region (Fig. 1, A). At his first neurological assessment performed ten months after surgery, a therapy with levetiracetam 1000 mg/day led to a decrease of the episodes, which completely disappeared after a dose increase to 1500 mg/day.

### Case 2

A 74-year-old woman experienced, in 2016, a cardiac surgery with aortic biological prosthesis placement and use of ECC. After a few weeks she started experiencing episodes with the following features: a cold sensation ascending rapidly from the lower limbs then turning into a sensation of heat rising from the chest to the face, accompanied by fear and bad smell, without loss of consciousness. The duration was a few minutes and the frequency was ranging from weekly to more than one per day. Two years after the cardiac surgery she performed a brain MRI revealing slight signs of chronic ischemic vascular suffering (Fig. 2, B), and a NAP-EEG showing spike during sleep in the right fronto-central-temporal derivation, and independent slow and sharp waves in the left fronto-temporal derivations (Fig. 1, B). Fifteen months after surgery, a pharmacologic treatment with levetiracetam was started, with disappearance of the episodes but occurrence of marked irritability. The patient stopped levetiracetam and the episodes started again, then a lamotrigine therapy was introduced (250 mg/day) with complete and persistent control of symptoms.

### Case 3

A 46-year-old man was operated on, in 2004, for mitral valve replacement with mechanical prosthesis with the support of ECC. Some weeks later he began suffering from peculiar episodes described as: a sudden onset of dream-like weird thoughts with slight anguish followed by intense nausea that lasted a few seconds and sometimes associated with an olfactory sensation not always unpleasant, without loss of consciousness. He also complained of a different type of disturbance, described as a sensation of double and blurred/dark vision prevailing the left part of the visual field. The frequency of each type of episode was variable from multi-monthly to multi-weekly, sometimes even daily or multi-daily. A brain MRI was performed fourteen years after the surgery, showing MBs in the right frontal region and in the right occipital pole (Fig. 2, C). NAP-EEG revealed sharp waves in the right parieto-occipital region and independent spikes localized in the left fronto-temporal derivations (Fig. 1, C). In January 2019 a pharmacologic treatment with levetiracetam was started, leading to a reduction in the duration and intensity of the episodes, nevertheless persisting at the same frequency, until June 2019 when alternative monotherapy with lacosamide up to 300 mg/day was introduced with almost complete disappearance of the episodes.

### Case 4

A 48-year-old man underwent, in 2012, a heart surgical intervention of ascending aorta replacement with aortic root graft using ECC. After five days he started complaining of episodes characterized by a tingling sensation starting from the right arm and spreading to the right leg, with a total duration of about 30–40 s, occurring at weekly/daily frequency. Two years after surgery he performed a brain MRI revealing an isolated MB in the left post-rolandic parietal region (Fig. 2, D), and the NAP-EEG showed sharp waves and spikes in the left fronto-parietal derivations during drowsiness and sleep (Fig. 1, D). Introduction of levetiracetam in 2014 until 2500 mg/day produced a complete remission of the episodes.

## Discussion and conclusions

We have observed a series of patients who, after a major cardiac surgery with ECC support, began to present frequent episodes with peculiar and homogeneous features, which raised the suspicion of an epileptic etiology. None of these patients had ever had convulsive seizures because of ischemic or hemorrhagic cerebral complications, events previously described following a heart surgery with ECC [1]. The episodes complained by our patients often continued for several years at high frequency, without being recognized. The EEG supported an epileptic origin, as well as the prompt and fairly complete response to antiepileptic therapy.

In most of our patients the MRI showed few lobar MBs in the subcortical brain regions (Table 1), some of which could be concordant with the clinical semiology of some episodes: two patients had episodes with visual semiology and evidence of MB in occipital areas while another one pointed out a somatosensory semiology with associated parietal MB. However, in other cases this relationship was not demonstrable: two experienced sensations indicating an involvement of the mesial temporal lobe region and two patients described features suggesting an insular origin of the epileptic discharge. Gustatory hallucinations associated with somatosensory sensation involving the face and the hands, together with a cold sensation in the lower limbs could suggest an insular lobe origin, as revealed by the intracranial electrical stimulation of the insula in epileptic patients [8]. Moreover, the same patient could have different kinds of episodes, and three patients had independent and asynchronous localization of the EEG abnormalities, suggesting a multifocal epilepsy. These aspects suggest that MB should probably be considered as a simple epiphenomenon, within a more complex and multifactorial circulatory disorder, which, in the presence of particular conditions, can become epileptogenic. Furthermore, MBs are widely described and often encountered in brain MRI of patients who underwent cardiac surgery [9], without epileptic seizures. Finally, since we do not have a pre-operative MRI study, we cannot exclude that MBs are a consequence of previous cardiac pathology. The potentially harmful effects of ECC on the cerebral circulation are usually associated with a risk of neurological complications such as stroke, bleeding, cognitive dysfunction, and delirium. The ECC flow is non or minimal pulsatile and the PaCO2 level is often higher than usual, negatively affecting the cerebral autoregulation [10, 11]. Furthermore, within such a short period of absent pulsatility, a heterogeneous flow in human capillaries ensues. It is marked by vessels with absent perfusion being closely adjacent to those with very fast perfusion [12]. This mechanism has been invoked in determining acute neurologic injury early expressed by seizures and myoclonus in the post cardiac arrest patient with or without extracorporeal cardiopulmonary resuscitation. In this context, hypocapnia-induced vasoconstriction might also play a certain role in the elicitation of seizures in susceptible patients immediately after global brain ischemia [13]. No medium-long term effects or late onset of neurological disorders on the epileptic side have been described up to now. This hypothesis could fit the pathophysiology of the events in the patients we have described and the isolated finding of MBs could be explained by the same mechanism, i.e. a mild and non-symptomatic form of hypoxic-ischemic brain damage by impaired cerebral autoregulation, without leukoencephalopathy as described in diffuse hypoxic-ischemic damage of critical illness [14].

**Table 1:** Clinical and neuroimaging features of the cases included in this study

	**Age, sex**	**Age at cardiac surgery**	**Follow-up (years)**	**Type of cardiac surgery**	**Time to seizure onset post-surgery**	**Seizure semiology**	**Seizure duration**	**Seizure frequency**	**NAP EEG**	**MRI**					**Therapy**	**Outcome**
										**Field Strength**	**GRE sequences**	**Microbleeds (number and location)**	**Leukoaraiosis (Fazekas score)**	**Cortical infarcts**		
**Case 1**	57, M	53	4	mitral valve reparation and ring annuloplasty	one week	1) bitter taste and perioral plus distal left hand fingers paresthesia 2) visual blackening in the left upper visual field quadrant	few second-max 1–2 min	Weekly/ monthly	1) spikes and slow waves in right PO 2) spikes and slow waves in left FT	1.5 T	SWI	3 lobar supratentorial MBs in the subcortical white matter of the right hemisphere	0	none	LEV 1500 mg/day	Seizure free
**Case 2**	74, W	71	3	aortic biological prosthesis placement	few weeks	cold sensation rapidly ascending from the lower limbs then turning in sensation of heat rising from the chest to the face, associated with fear and bad smell	few minutes	Weekly/daily/multidaily	1) spikes in right FCT 2) slow and sharp waves in left FT	1.5 T	SWAN	0	1	none	LTG 250 mg/die	Seizure free
**Case 3**	46, M	32	14	mitral valve replacement with mechanical prosthesis	some weeks	1) sudden onset of dream-like weird thought with slight anguish followed by intense nausea during few seconds and sometimes associated with olfactory sensation not always unpleasant 2) double and blurred/dark vision prevailing the left part of the visual field	few seconds	Monthly/weekly/daily/multidaily	1) sharp waves in right PO 2) spikes in left FT	1.5 T	T2*	4 lobar supratentorial MBs (3 in the subcortical white matter of the right frontal lobe and 1 cortical right occipital)	0	none	LCS 300 mg/die	Marked reduction in frequency and duration
**Case 4**	48, M	41	7	ascending aorta replacement with aortic root graft	five days	tingling sensation starting from the right arm and spreading to the right leg	30–40 s	weekly/daily	sharp waves and spikes in left FP	1.5 T	T2*	1 lobar supratentorial MB	0	none	LEV 2500 mg/day	Seizure free

*Abbreviations*: *NAP EEG* EEG during sleep, *FT *Fronto-temporal, *FCT *Fronto-central-temporal , *GRE *Gradient echo, *LEV *Levetiracetam, *LTG *Lamotrigine, *LCS* Lacosamide, *MRI *Magnetic Resonance Imaging, *MBs* Microbleeds, *PO *parieto-occipital, *SWI* Susceptibility weighted imaging, *SWAN* Susceptibility-weighted angiography

However, it is important to recognize that such MB can be observed in patients with ECMO, or even without ECMO (for example hypertension, cerebral amyloidosis, CNS vasculitis) without any definite clinical correlation. Limited data exists regarding the epidemiology of seizures in adults after cardiac surgery, and particularly lacking is data on epilepsies. The great majority of postoperative seizures reported in literature are in the form of generalized tonic–clonic convulsions, or seizures with clinically apparent signs and symptoms, which are readily recognized by the observers. Nevertheless, a large proportion of non-convulsive seizure (NCS) exists, ranging from 8 and 40% in critically ill patients [15–17], which are difficult to detect without performing an EEG. The few studies focusing on seizures in adults after cardiac surgery have considered generalized tonic–clonic seizures (81% in Manji et al. [18], 71% in Goldstone et. al. [5]), simple/complex partial seizure (26% in Goldstone et. al. [5]), or status epilepticus (8% in Manji et al. [18], 3% Goldstone et al. [5]), without specific characterization of the type of partial seizures. Seizures with subjective manifestations, such as those described in our cases, have never been studied, so their frequency is unknown. Most postoperative seizures reported in literature occurred in the first hours or few days after surgery, and often secondary to complications such as ischemic stroke, so constituting the condition of acute symptomatic seizure [19]. Moreover, according to Manji et. al, 89% of patients who experienced recurrence of seizures had them within 24 h of the first seizures, so representing an expression of the same brain insult [18]. Thus, patients who develop epilepsy are a minority (only 11% of patients reported by Manji et. al with recurrent seizures experienced beyond 24 h from the first seizures), and studies focused on this topic are missing in the literature [18].

In conclusion, we describe a series of patients developing epilepsy with similar characteristics after cardiac surgery using ECC. In particular, in our cases, the seizures’ semiology suggests an involvement of the insular, temporal, parietal or occipital lobe. Moreover, we observed that the seizures are often of a multifocal origin, that they occur with high frequency but low impact on daily life and without loss of consciousness or motor manifestations, and that the response to antiepileptic therapy is prompt and persistent. Because of their features, these kinds of seizures tend to be often under-recognized, leading to a frequent delay in the start of treatment and possibly alteration of neuronal networks with the increasing risk of a convulsive seizure. However, the features of the epilepsies reported in this short case series cannot be generalized to the entire population of patients presenting seizures and epilepsies following cardiac surgery with ECC, and future prospective and systematic studies are needed to better define the possible correlation between ECC and specific seizure types.

## Data Availability

Data sharing is not applicable to this article as no datasets were generated or analysed during the current study.

## Abbreviations

DWI: Diffusion-weighted imaging
ECC: ExtraCorporeal Circulation
ECMO: Extracorporeal membrane oxygenation
EEG: Electroencephalogram
EEG-NAP: Electroencephalogram during sleep
MB: Microbleed
MBs: Microbleeds
MRI: Magnetic resonance imaging
